# Modeling of wheelchair movement trajectory error and analysis of self-correcting control mechanism in disabled table tennis

**DOI:** 10.1371/journal.pone.0324353

**Published:** 2025-06-05

**Authors:** Qibin Feng, Jiaju Li, Zhengyu Su

**Affiliations:** 1 School of Physical Education, Suihua University, Suihua, Heilongjiang, China; 2 School of Special Education, Suihua University, Suihua, Heilongjiang, China; 3 College of Physical Education, Liaoning Normal University, Dalian, Liaoning, China; Pennsylvania State University, UNITED STATES OF AMERICA

## Abstract

A wheelchair kinematic model was constructed to address the wheelchair control problem in table tennis for disabled individuals. The influence of slipping factors was considered, and a differential model of motion trajectory error was derived. A table tennis wheelchair self-correcting control model was designed based on the back-stepping method. Under the self-correcting control model, the state observer continuously observes the error of the motion trajectory and provides compensation to suppress disturbances including slippage, ensuring the stability of the table tennis wheelchair operation. The Lyapunov stability of the proposed self-correcting control model has been demonstrated. Through two sets of simulation experiments, the control effect of the self-correcting model on the linear and circular movement of the wheelchair, especially the suppression effect on slipping interference, was verified. The comparative experimental results show that under the control of the self-correcting model, the error variation of the wheelchair’s movement trajectory is significantly smaller than that of the PID method and PSO method.

## 1 Introduction

The 2024 Paris Paralympic Games once again witnessed the social value of people with disabilities. Wheelchair table tennis is an important competition event in disabled sports [1]. As early as the first Paralympic Games in 1960, wheelchair table tennis was included in the official competition. In this sport, the wheelchair will move according to the athlete’s movement trend to assist the athlete in hitting the ball. With the increasing maturity of modern control technology, installing controllers in wheelchairs to elevate their passive movement to adaptive movement can better ensure the correctness of athletes’ poses.

The automatic control transformation of table tennis wheelchairs belongs to the research scope of intelligent wheelchairs, and intelligent wheelchairs have many similarities with four-wheel mobile robots [2–3]. To ensure the mobility and control accuracy of the table tennis wheelchair, it is necessary to mathematically model the wheelchair itself and set up a reasonable controller [4]. The kinematic equations of a wheelchair reflect the geometric relationship between the rotational speed of each wheel and the overall movement speed, while the dynamic equations of a wheelchair reflect the physical relationship between power and motion state [5–6].

Tache derived and established a kinematic model using vector analysis method, which has good applicability to wheeled robots [7]. Félix established kinematic and dynamic equations using the spinor method and Newton Euler method, respectively, by analyzing the mechanical structure relationship of the wheelchair [8]. Fu derived the wheelchair motion equation at any angle based on the fundamental principles of nonholonomic mechanics [9]. Rotondo conducted kinematic and dynamic modeling of a four-wheel robot and analyzed the effects of friction, external forces, and uncertainty on the motion equations [10]. Takagi analyzed the motion process of mobile robots and used nonlinear predictive control to achieve robot trajectory tracking [11]. Hernandez proposed a friction compensated predictive control algorithm that reduces the impact of friction on the trajectory of mobile robots [12]. Samodro proposed a sliding mode robust adaptive control algorithm that suppresses the impact of interference and uncertainty on the efficiency of robot movement [13]. Sepulveda Valdez proposed a neural network control method based on recursive fuzzy wavelets, which improved the robustness of mobile robot control [14]. Mai designed a variable structure fuzzy control scheme based on fuzzy control, which improved the control performance of mobile robots [15]. Mercorelli has conducted a series of research works on the control problems of wheeled robots, and has successively proposed control integrated MPC technology based on flatness and controller based on cascaded fuzzy, which have improved the dynamic performance and control effect of non integral wheeled mobile robots [16–18]. Hernandez used the Mecanum wheeled mobile robot as an example to develop a two-level controller, where the low-level controller is responsible for adjusting the speed of the wheels and the high-level controller is responsible for the robot’s movement position. This two-level controller has achieved good results, enabling the robot to have higher maneuverability [19]. Samodro’s control of mobile robots is based on the artificial potential field method, which achieves effective obstacle avoidance by driving the robot’s position through differential kinematics equations without collision targets [20, 21].

The kinematic and control model research of mobile robots, especially wheeled robots, by scholars is of great significance for the effective control of table tennis wheelchairs. In this article, we will conduct kinematic analysis and control method research on electric table tennis wheelchairs. In the future, the control method proposed in this article will be applied to an electric table tennis wheelchair. This electric table tennis wheelchair will integrate sensors, controllers, and the wheelchair mechanical body. The sensors are responsible for collecting the athlete’s center of gravity position, force, and other information. The control method proposed in this article runs on the controller to achieve automatic control and adjustment of the wheelchair. In the research work of this article, in addition to drawing on previous research ideas, the influence of some special issues in table tennis wheelchair sports is also considered. During exercise, excessive deviation of the athlete’s center of gravity and wear on the tires and ground can lead to a decrease in friction, causing slippage between the wheelchair and the ground, which in turn affects the accurate movement of the wheelchair and the athlete’s ability to return the ball and exert force. In this article, taking the problem of slipping as the starting point, the kinematic equations and differential equations of the running trajectory error of the table tennis wheelchair are constructed, and a control model with compensation mechanism is designed to prove its Lyapunov stability and verify it through simulation experiments.

## 2 Establishment of mathematical model for table tennis wheelchair

### 2.1 Kinematic modeling of table tennis wheelchair

**Fig 1:**
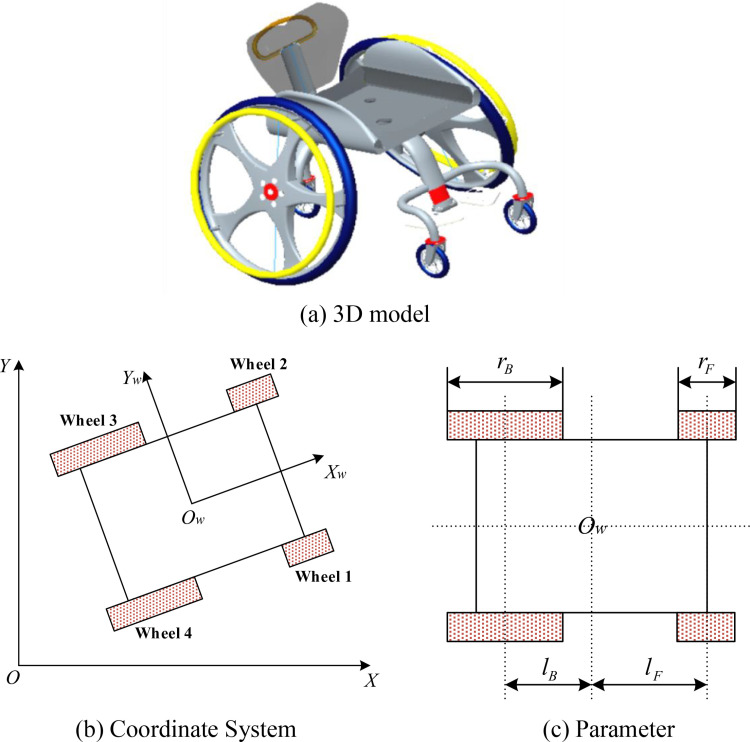
Structural of table tennis wheelchair. (a) 3D model b) Coordinate System (c) Parameter.

In table tennis for disabled people, the movement of athletes’ positions relies on wheelchairs. This wheelchair is specifically designed for disabled athletes, with the two large wheels at the bottom being the main moving components, and the other two small wheels used to coordinate the movement of the large wheels. The structure of this wheelchair is shown in Fig 1.

Due to its special structural design, both the large and small wheels in the wheelchair can move in all directions. In order to facilitate the description of the motion state of the wheelchair, the world coordinate system O(X,Y) and wheelchair coordinate system $O_{w}(X_{w},Y_{w})$ are set separately. Under normal conditions, where there is sufficient friction between the ground and the wheels and the wheels do not slip, the kinematic model of the wheelchair is shown in formula (1):


$$\begin{array}{c} \left\{ \begin{array}{c} {\dot{X}}_{w}=-\frac{1}{4}r_{1}\omega_{1}+\frac{1}{4}r_{2}\omega_{2}-\frac{1}{4}r_{3}\omega_{3}+\frac{1}{4}r_{4}\omega_{4}\\ {\dot{Y}}_{w}=\frac{1}{4}r_{1}\omega_{1}+\frac{1}{4}r_{2}\omega_{2}+\frac{1}{4}r_{3}\omega_{3}+\frac{1}{4}r_{4}\omega_{4}\\ {\dot{\varphi }}_{w}=\frac{r_{1}\omega_{1}}{4(l_{B}+l_{F})}-\frac{r_{2}\omega_{2}}{4(l_{B}+l_{F})}-\frac{r_{3}\omega_{3}}{4(l_{B}+l_{F})}+\frac{r_{4}\omega_{4}}{4(l_{B}+l_{F})} \end{array}\right.\; \end{array}$$ (1)

Here, ${\dot{X}}_{w}$ is the *X*-direction velocity of the wheelchair in the wheelchair coordinate system, ${\dot{Y}}_{w}$ is the *Y*-direction velocity of the wheelchair in the wheelchair coordinate system, and ${\dot{\varphi }}_{w}$ is the rotational speed of the wheelchair around the center of the wheelchair coordinate system. $r_{i}(i=1,2,3,4)$ is the radius of the *i*-th wheel, where $r_{1}=r_{2}=r_{F}$($r_{F}$ is the radius of the front wheel) and $r_{3}=r_{4}=r_{B}$ ($r_{B}$ is the radius of the rear wheel). $\omega_{i}(i=1,2,3,4)$ is the angular velocity of the *i*-th wheel. $l_{F}$ is the distance between the centerline of the two front wheels and the center of the wheelchair coordinate system, and $l_{B}$ is the distance between the centerline of the two rear wheels and the center of the wheelchair coordinate system.

### 2.2 Kinematic modeling of wheelchair under the influence of slipping

During the actual competition of wheelchair table tennis, there may be situations where the wheelchair slips due to various reasons such as aging wheel surfaces, overly smooth ground. When a wheelchair slips, the athlete’s power and movement will be affected, and in severe cases, it can cause injury. Therefore, if a compensation mechanism can be established and the wheelchair’s movement can be automatically adjusted through a controller to counteract the effects of slipping, it can better protect athletes and promote the healthy development of table tennis for people with disabilities.

Therefore, it is necessary to further consider the kinematic modeling of wheelchairs under the influence of slippage based on formula (1). If the *i*-th wheel in the wheelchair slips, its slip rate is calculated as follows:


$$\delta_{i}=\frac{r_{i}\omega_{i}-v_{i}^{\delta }}{r_{i}\omega_{i}}$$ (2)

Here, $\delta_{i}$ is the slip rate of the *i*-th wheel in the wheelchair, $r_{i}(i=1,2,3,4)$ is the radius of the *i*-th wheel, $\omega_{i}(i=1,2,3,4)$ is the angular velocity of the *i*-th wheel, and $v_{i}^{\delta }$ is the linear velocity of the *i*-th wheel relative to the ground when slipping.

Based on this, a wheelchair kinematic model representing the line velocity in a slipping state can be derived, as shown in formula (3):


$$\begin{array}{c} \left\{ \begin{array}{c} {\dot{X}}_{w}=-\frac{1}{4}v_{1}^{\delta }+\frac{1}{4}v_{2}^{\delta }-\frac{1}{4}v_{3}^{\delta }+\frac{1}{4}v_{4}^{\delta }\\ {\dot{Y}}_{w}=\frac{1}{4}v_{1}^{\delta }+\frac{1}{4}v_{2}^{\delta }+\frac{1}{4}v_{3}^{\delta }+\frac{1}{4}v_{4}^{\delta }\\ {\dot{\varphi }}_{w}=\frac{v_{1}^{\delta }}{4(l_{B}+l_{F})}-\frac{v_{2}^{\delta }}{4(l_{B}+l_{F})}-\frac{v_{3}^{\delta }}{4(l_{B}+l_{F})}+\frac{v_{4}^{\delta }}{4(l_{B}+l_{F})} \end{array}\right.\; \end{array}$$ (3)

Furthermore, a wheelchair kinematic model representing angular velocity in a slipping state can be derived, as shown in formula (4):


$$\begin{array}{c} \left\{ \begin{array}{c} {\dot{X}}_{w}=-\frac{1}{4}r_{1}\omega_{1}(1-\delta_{1})+\frac{1}{4}r_{2}\omega_{2}(1-\delta_{2})-\frac{1}{4}r_{3}\omega_{3}(1-\delta_{3})+\frac{1}{4}r_{4}\omega_{4}(1-\delta_{4})\\ {\dot{Y}}_{w}=\frac{1}{4}r_{1}\omega_{1}(1-\delta_{1})+\frac{1}{4}r_{2}\omega_{2}(1-\delta_{2})+\frac{1}{4}r_{3}\omega_{3}(1-\delta_{3})+\frac{1}{4}r_{4}\omega_{4}(1-\delta_{4})\\ {\dot{\varphi }}_{w}=\frac{r_{1}\omega_{1}(1-\delta_{1})}{4(l_{B}+l_{F})}-\frac{r_{2}\omega_{2}(1-\delta_{2})}{4(l_{B}+l_{F})}-\frac{r_{3}\omega_{3}(1-\delta_{3})}{4(l_{B}+l_{F})}+\frac{r_{4}\omega_{4}(1-\delta_{4})}{4(l_{B}+l_{F})} \end{array}\right.\; \end{array}$$ (4)

Considering the conversion relationship between the world coordinate system and the wheelchair coordinate system, there exists the following form:


$$\;\left[\begin{array}{c} X\\ Y\\ \varphi \end{array}\right]=[\begin{array}{ccc} \cos\varphi & -\sin\varphi & 0\\ \sin\varphi & \cos\varphi & 0\\ 0 & 0 & 1 \end{array}]\left[\begin{array}{c} X_{w}\\ Y_{w}\\ \varphi_{w} \end{array}\right]$$ (5)

Thus, the kinematic model of the wheelchair in the world coordinate system can be obtained, as shown in formula (6):


$$\begin{array}{c} \left\{ \begin{array}{c} \dot{X}={\dot{X}}_{w}\cos\varphi -{\dot{Y}}_{w}\sin\varphi \\ \dot{Y}={\dot{X}}_{w}\sin\varphi +{\dot{Y}}_{w}\cos\varphi \\ \dot{\varphi }={\dot{\varphi }}_{w} \end{array}\right.\; \end{array}$$ (6)

By substituting formula (4) into formula (6), a kinematic model based on linear velocity of the wheelchair in the world coordinate system during slipping can be obtained. By substituting formula (5) into formula (6), the kinematic model of the wheelchair in the world coordinate system based on angular velocity during slipping can be obtained.

### 2.3 Modeling of motion trajectory error for table tennis wheelchair

In order to achieve effective control of the table tennis wheelchair, a reasonable error model needs to be established to better track the trajectory of the wheelchair’s movement. In the world coordinate system, set the actual pose information of the wheelchair to $[X,Y,\varphi {]}^{T}$, and set the expected pose information of the wheelchair to ${\left[X_{e},Y_{e},\varphi_{e}\right]}^{T}$. So, the pose error model of the table tennis wheelchair is shown in formula (7):


$$\begin{array}{c} \left\{ \begin{array}{c} \eta_{x}=\cos\varphi (X_{e}-X)+\sin\varphi (Y_{e}-Y)\\ \eta_{y}=-\sin\varphi (X_{e}-X)+\cos\varphi (Y_{e}-Y)\\ \eta_{\varphi }=\varphi_{e}-\varphi \end{array}\right.\; \end{array}$$ (7)

Here, $\eta_{x}$ is the *X*-axis position error of the wheelchair in the moving coordinate system, $\eta_{y}$ is the *Y*-axis position error of the wheelchair in the moving coordinate system, and $\;\eta_{\varphi }$ is the body angle error of the wheelchair in the moving coordinate system.

Also, the differential equation for the expected pose of a table tennis wheelchair is as follows:


$$\begin{array}{c} \;\left\{ \begin{array}{c} {\dot{X}}_{e}=v_{e}\cos\varphi_{e}\\ {\dot{Y}}_{e}=v_{e}\sin\varphi_{e}\\ {\dot{\varphi }}_{e}=\omega_{e} \end{array}\right.\; \end{array}$$ (8)

Here, $v_{e}$ is the expected linear velocity, and $\omega_{e}$ is the expected angular velocity of the wheelchair as a whole.

By combining formulas (7) and (8), the differential equation for the pose error model of the table tennis wheelchair can be obtained, as shown in formula (9):


$$\begin{array}{c} \;\left\{ \begin{array}{c} {\dot{\eta }}_{x}=\omega \eta_{y}+v_{e}\cos\eta_{\varphi }-v_{x}\\ {\dot{\eta }}_{y}=-\omega \eta_{x}+v_{e}\sin\eta_{\varphi }-v_{y}\\ {\dot{\eta }}_{\varphi }=\omega_{e}-\omega \end{array}\right.\; \end{array}$$ (9)

Here, $v_{x}$ is the *X*-axis component of the actual linear velocity of the wheelchair, $v_{y}$ is the *Y*-axis component of the actual linear velocity of the wheelchair, and ω is the actual angular velocity of the wheelchair.

## 3 Controller design and mechanism analysis of table tennis wheelchair

### 3.1 Controller model design

**Fig 2:**
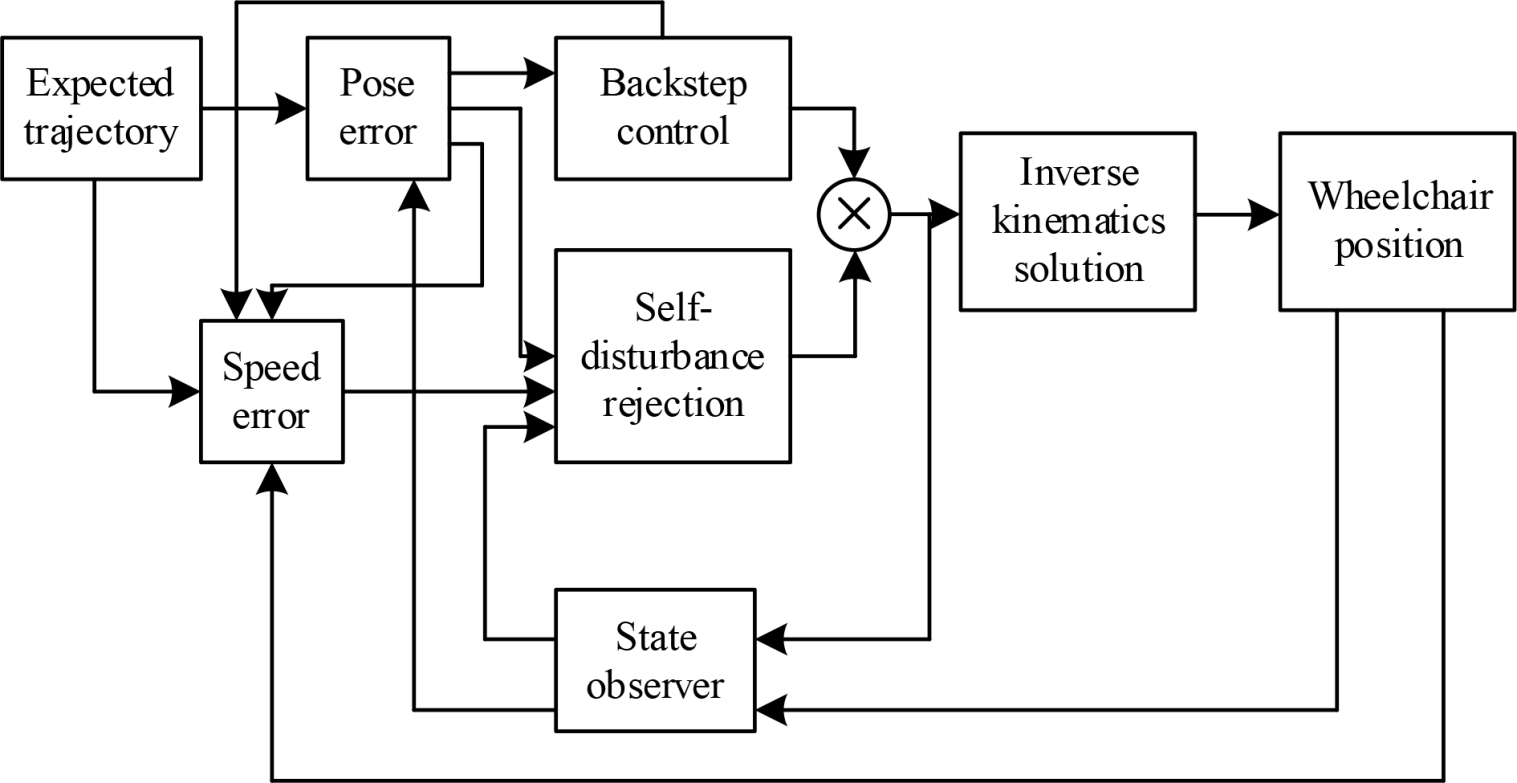
Overall design framework of the controller.

In order to achieve reliable control of the table tennis wheelchair, starting from the expected position of the wheelchair posture, the mathematical model of the controller is constructed step by step according to the backstepping method, combined with the error feedback model shown in formula (7). The basic idea here is to decompose complex nonlinear systems into subsystems that do not exceed the highest order of the system. Then, design Lyapunov functions and intermediate virtual control variables for each subsystem, gradually extrapolate to the entire system, and complete the design of the entire controller. This method obtains a feedback controller by recursively constructing a Lyapunov function, and selects a control law to make the derivative of the Lyapunov function along the trajectory of the closed-loop system have certain performance, ensuring the boundedness of the closed-loop system trajectory and convergence to the equilibrium point. Here, the overall design framework of the controller is presented, as shown in Fig 2.

In Fig 2, the input of the controller includes the current motion information of the wheelchair, such as position, posture, speed, etc. Because the subsequent experiments were conducted in a simulation environment, the expected trajectory information was also included at the input end. In the future, when the controller is applied to an electric wheelchair, the motion status information and trajectory trend information of the wheelchair will be automatically obtained through sensors.

Here, first of all, without considering the situation of wheel slippage, the pose and error of the wheelchair during the control process satisfy the Lyapunov function, as shown in formula (10).


$$\;U(t)=\frac{1}{2}\eta_{x}^{2}+\frac{1}{2}\eta_{y}^{2}+\frac{1-\cos\eta_{\varphi }}{\lambda_{1}}$$ (10)

Here, U(t) is a Lyapunov function, and $\lambda_{1}$ is a positive constant that can be adjusted.

By taking the derivative of this Lyapunov function, we can obtain:


$$\;\dot{U}(t)=\eta_{x}{\dot{\eta }}_{x}+\eta_{y}{\dot{\eta }}_{y}+\frac{\sin\eta_{\varphi }}{\lambda_{1}}{\dot{\eta }}_{\varphi }$$ (11)

By substituting the differential equation of the wheelchair pose error model represented by formula (9) into formula (11), we can obtain:


$$\;\dot{U}(t)=\eta_{x}(\omega \eta_{y}+v_{e}\cos\eta_{\varphi }-v_{x})+\eta_{y}(-\omega \eta_{x}+v_{e}\sin\eta_{\varphi }-v_{y})+\frac{\lambda_{2}\sin\eta_{\phi }}{\lambda_{1}}(\dot{\varphi }-\omega )$$ (12)

Furthermore, the kinematic control model of the table tennis wheelchair can be obtained, as shown in formula (13):


$$\begin{array}{c} \;\left\{ \begin{array}{c} v_{x}=v_{e}\cos\eta_{\varphi }+\lambda_{4}\eta_{x}\\ v_{y}=v_{e}\sin\eta_{\varphi }+\lambda_{3}\eta_{y}\\ v_{\varphi }=\omega_{e}+\lambda_{2}\sin\eta_{\varphi } \end{array}\right.\; \end{array}$$ (13)

Here, $\lambda_{1}$, $\lambda_{2}$, $\lambda_{3}$, and $\lambda_{4}$ are all adjustable positive constants. And they establish the following relationship:


$$\;\dot{U}(t)=-\lambda_{4}\eta_{x}^{2}-\lambda_{3}\eta_{y}^{2}-\frac{\lambda_{2}}{\lambda_{1}}\sin^{2}\eta_{\varphi }\leq 0$$ (14)

According to the inverse kinematics solution of the table tennis wheelchair model, the rotational speeds of the four wheels can be obtained, as shown in formula (15):


$$\begin{array}{c} \;\left\{ \begin{array}{c} \omega_{1}=\frac{1}{r_{1}}\left[-v_{x}+v_{y}+(l_{B}+l_{F})v_{\varphi }\right]\\ \omega_{2}=\frac{1}{r_{2}}\left[v_{x}+v_{y}-(l_{B}+l_{F})v_{\varphi }\right]\\ \omega_{3}=\frac{1}{r_{3}}\left[-v_{x}+v_{y}-(l_{B}+l_{F})v_{\varphi }\right]\\ \omega_{4}=\frac{1}{r_{4}}\left[v_{x}+v_{y}+(l_{B}+l_{F})v_{\varphi }\right] \end{array}\right.\; \end{array}$$ (15)

Let’s consider the situation where the wheels of the wheelchair slip. In order to maintain the stability of the wheelchair, a state observer can be constructed to estimate and compensate for the interference during sliding in real time. The state observer for slip interference is shown in formula (16):


$$\begin{array}{c} \;\left\{ \begin{array}{c} {\hat{v}}_{x}=\rho_{x}(\delta_{i},\omega ,\varphi ,t)+b_{1}v_{x}\\ {\hat{v}}_{y}=\rho_{y}(\delta_{i},\omega ,\varphi ,t)+b_{2}v_{y}\\ {\hat{v}}_{\varphi }=\rho_{\varphi }(\delta_{i},\omega ,\varphi ,t)+b_{3}v_{\varphi } \end{array}\right.\; \end{array}$$ (16)

Here, ${\hat{v}}_{x}$ is the estimated value of $v_{x}$, $\;\rho_{x}(\delta_{i},\omega ,\omega ,t)$ is the interference function of slipping to $v_{x}$, and it is related to parameters such as slipping rate, wheelchair angular velocity, wheelchair angle, time, etc. ${\hat{v}}_{y}$ is the estimated value of $v_{y}$, $\;\rho_{y}(\delta_{i},\omega ,\varphi ,t)$ is the interference function of the slipping to *v*_*y*_. ${\hat{v}}_{\varphi }$ is the estimated value of $\;v_{\varphi }$, $\;\rho_{\varphi }(\delta_{i},\omega ,\varphi ,t)$ is the interference function of the slipping to $\;v_{\varphi }$. $b_{1}$, $b_{2}$, and $b_{3}$ are all adjustable coefficient.

When there is slip interference, by compensating with the state observer and satisfying the following formula, effective tracking and stable control of the wheelchair trajectory can be achieved, and the influence of slip error can be eliminated.


$$\;\lim_{t\to \infty }\left\|[\begin{array}{ccc} \eta_{x} & \eta_{y} & \eta_{\varphi } \end{array}]T\right\|=0$$ (17)

### 3.2 Stability proof of controller

When slipping, considering the compensation control of the state observer, the trajectory error differential model of the table tennis wheelchair will change from formula (9) to formula (18):


$$\begin{array}{c} \;\left\{ \begin{array}{c} {\dot{\eta }}_{x}=(\omega +\rho_{\varphi })\eta_{y}+v_{e}\cos\eta_{\varphi }-(v_{x}+\rho_{x})\\ {\dot{\eta }}_{y}=-(\omega +\rho_{\varphi })\eta_{x}+v_{e}\sin\eta_{\varphi }-(v_{y}+\rho_{y})\\ {\dot{\eta }}_{\varphi }=\omega_{e}-(\omega +\rho_{\varphi }) \end{array}\right.\; \end{array}$$ (18)

**Theorem:** In the slipping state, the table tennis wheelchair is a Lyapunov stable system under the control model shown in formula (13). When t→∞, the trajectory tracking error $\eta_{x}$, $\eta_{y}$, and $\eta_{\varphi }$ of the table tennis wheelchair asymptotically converges to 0.

**Proof:** The kinematic control process of the table tennis wheelchair selected the Lyapunov function shown in formula (10), that is $U(t)=\frac{1}{2}\eta_{x}^{2}+\frac{1}{2}\eta_{y}^{2}+\frac{1-\cos\eta_{\varphi }}{\lambda_{1}}$. And its differential form is shown in formula (11), that is $\;\dot{U}(t)=\eta_{x}{\dot{\eta }}_{x}+\eta_{y}{\dot{\eta }}_{y}+\frac{\sin\eta_{\varphi }}{\lambda_{1}}{\dot{\eta }}_{\varphi }$. For any $\;{\left[\begin{array}{ccc} \eta_{x} & \eta_{y} & \eta_{\varphi } \end{array}\right]}^{T}\neq 0$, U(t)>0.

When slipping, substituting formula (19) into the differential equation of the Lyapunov function yields:


$$\begin{array}{c} \;\dot{U}(t)=\begin{array}{c} \eta_{x}\left[(\omega +\rho_{\varphi })\eta_{y}+v_{e}\cos\eta_{\varphi }-(v_{x}+\rho_{x})\right]\\ \eta_{y}\left[-(\omega +\rho_{\varphi })\eta_{x}+v_{e}\sin\eta_{\varphi }-(v_{y}+\rho_{y})\right]\\ \frac{\lambda_{2}\sin\eta_{\varphi }}{\lambda_{1}}\left[\dot{\varphi }-(\omega +\rho_{\varphi })\right] \end{array} \end{array}$$ (19)

By substituting the control model shown in formula (13) into the differential equation shown in formula (20) and combining similar terms, we can obtain:

$\dot{U}(t)=-\kappa_{1}\eta_{x}^{2}-\kappa_{2}\eta_{y}^{2}-\kappa_{3}\sin^{2}\eta_{\varphi }$ (20)

Here, $\kappa_{1}$, $\kappa_{2}$, and $\kappa_{3}$ are normal number greater than 0, so $\dot{U}(t)\leq 0$.Therefore, in the slipping state, the table tennis wheelchair is a monotonically bounded Lyapunov stable system under the control model shown in formula (13). Therefore, in the slipping state, the Lyapunov function shown in formula (10) is monotonically bounded. In the case where the expected posture of a wheelchair is bounded, ${\dot{\eta }}_{x}$, ${\dot{\eta }}_{y}$, and $\;{\dot{\eta }}_{\kappa }$ is bounded. According to Barbalat’s lemma, when t→∞, the trajectory tracking error $\eta_{x}$, $\eta_{y}$, and $\eta_{\varphi }$ of the table tennis wheelchair asymptotically converges to 0.

## 4 Simulation analysis of self-correcting control for table tennis wheelchair

In the previous work, research was conducted on wheelchair control issues in table tennis for people with disabilities. A wheelchair kinematic model was constructed, taking into account the influence of slip factors, and a differential model of motion trajectory error was derived. A table tennis wheelchair self correcting control model was designed based on the backstepping method. Under the self correcting control model, the state observer continuously observes the error of the motion trajectory and provides compensation to suppress disturbances including slippage, ensuring the stability of the table tennis wheelchair operation. The Lyapunov stability of the proposed self correcting control model has been demonstrated. In this part of the work, the kinematic model, trajectory error differential model, and self-tuning control model of the table tennis wheelchair will be validated through simulation analysis.

During the process of table tennis for disabled people, the wheelchair will move according to the athlete’s movement trend. Wheelchairs may have various complex movement trajectories, but these trajectories are composed of basic linear and circular movements. Therefore, the simulation experiment is divided into two groups. The first group is the simulation of the control effect of the table tennis wheelchair moving along a straight trajectory, and the first group is the simulation of the control effect of the table tennis wheelchair moving along a circular trajectory.

During the simulation experiment, the two front wheel radii of the table tennis wheelchair were set to $r_{1}$=$r_{2}$=0.05m, and the two rear wheel radii of the table tennis wheelchair were set to $r_{3}$=$r_{4}$=0.3m. Meanwhile, set two additional parameters $l_{F}$= 0.4m and $l_{B}$= 0.2m.

### 4.1 Simulation analysis of table tennis wheelchair moving along a straight trajectory

In the experiment of linear trajectory movement, the table tennis wheelchair is set to move along the diagonal of the field, that is, the coordinate system pose of the wheelchair satisfies: *X* = *t*, *Y* = *t*,  φ = 0. The movement speed of the table tennis wheelchair in the *X* and *Y* directions is set to 1m/s, and its initial position is set to *X* = -3, *Y* = 2,  φ = 0. The starting point of the linear trajectory is, *X* = 0, *Y* = 0.

The table tennis wheelchair is affected by slipping during movement. The two wheels on the right side of the wheelchair have slipped, with a slip time set at *t* = 0.5 ~ 1.0s. Within this time range, the slip rate of the right front wheel is $\delta_{1}$=0.6, and the slip rate of the right rear wheel is $\delta_{4}$=0.1.

**Fig 3:**
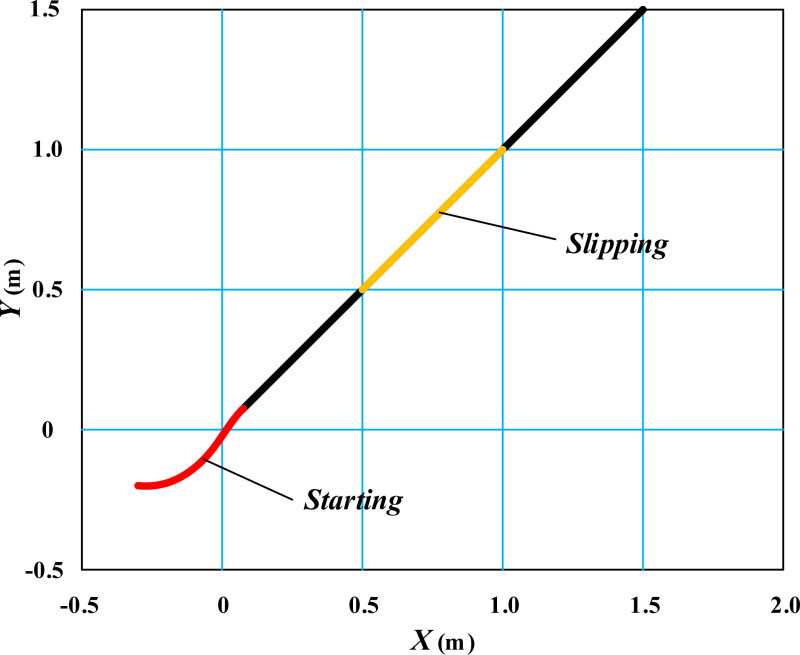
Simulation results of wheelchair linear trajectory movement under self correction control.

The simulation results of the movement trajectory of the table tennis wheelchair on the field under the proposed self correcting control model are shown in Fig 3.

From Fig 3, it can be seen that under the control of the proposed self correcting model, the table tennis wheelchair smoothly cuts into a straight trajectory from its initial position and moves smoothly along the diagonal straight trajectory. Within the time range of *t* = 0.5 ~ 1.0s, there is a slipping effect between the ground and the wheelchair, but under the control of the self correcting model, the movement trajectory of the wheelchair remains stable. In Fig 3, the starting position of the table tennis wheelchair is not selected at the origin, considering that there will be some disturbance during the initial movement when the wheelchair is electrically controlled. It is necessary to make brief adjustments under the algorithm control of the controller to enter the expected straight trajectory.

**Fig 4:**
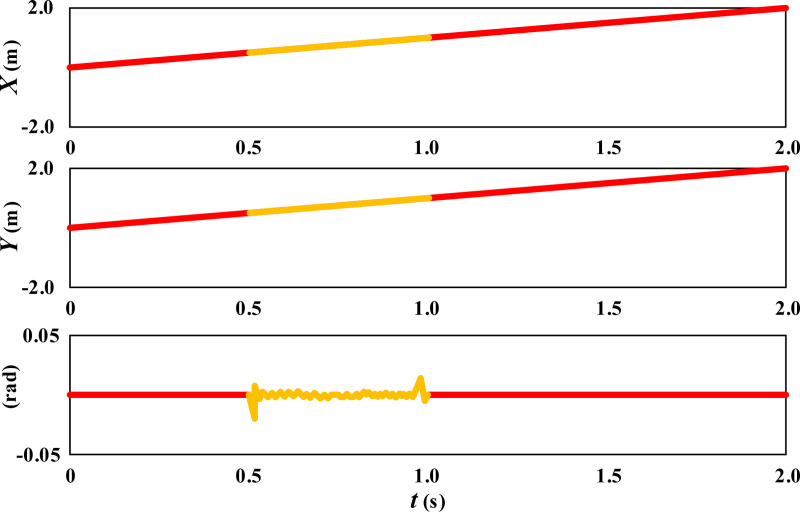
Dimensionality expansion results of the straight-line movement posture of the wheelchair.

Expand the linear movement posture of the wheelchair in Fig 3 along the time axis, as shown in Fig 4.

From the results in Fig 4, it can be seen that during the process of linear movement, the wheelchair maintains a linear change in both the X and Y directions. Although the slip of 0.5-1s has a slight impact, the self correcting control model can provide the fastest correction, ensuring the stability of the wheelchair movement trajectory. During the process of moving in a straight line, the angle of the wheelchair remains basically straight, that is, the posture is stable. The 0.5-1s slip caused a trend of angle adjustment for the wheelchair, but the self correcting model was able to provide timely correction, ensuring the stability of the wheelchair angle.

**Fig 5:**
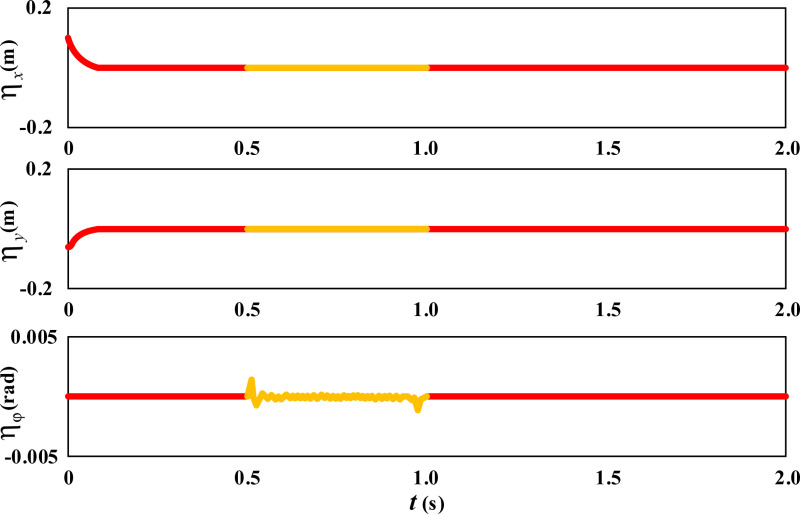
Dimensionality Expansion Results of Linear Movement Error of Wheelchair.

Further observe the error changes in the movement trajectory of the wheelchair during the movement process, as shown in Fig 5.

From Fig 5, it can be seen that during linear movement, the trajectory error of the wheelchair in both the *X* and *Y* directions is basically zero. Only during a short period of time from the initial position to entering a straight trajectory, the wheelchair has a certain degree of movement error due to adjusting its posture to ensure correct entry into the diagonal. Afterwards, the wheelchair continued to move diagonally, and the trajectory errors in the X and Y directions disappeared. The angle error $\eta_{\varphi }$ of the wheelchair fluctuates slightly between 0.5 and 1 second, which is the result of the wheelchair continuously correcting the angle to cope with slipping.

### 4.2 Simulation analysis of table tennis wheelchair moving along a circular trajectory

In the circular trajectory movement experiment, the table tennis wheelchair is set to move along a circular trajectory. The center of the circular trajectory is point (0,0) with a radius of 1. The coordinate system pose of the wheelchair satisfies: *X* = sin*t*, *Y* = cos*t*, φ =0. The starting point of the circular trajectory of the table tennis wheelchair is *X* = 0, *Y* = 0.

The table tennis wheelchair is affected by slipping during movement. The two wheels on the right side of the wheelchair have slipped, with a slip time set at t = 0.5 ~ 1.0s. Within this time range, the slip rate of the right front wheel is $\delta_{1}$=0.6, and the slip rate of the right rear wheel is $\delta_{4}$=0.1.

**Fig 6:**
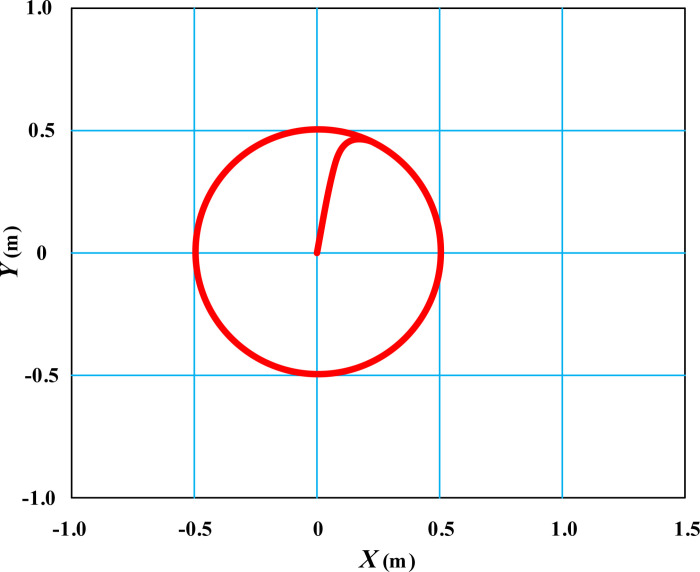
Simulation results of wheelchair circular trajectory movement under self correction control.

The simulation results of the movement trajectory of the table tennis wheelchair on the field under the proposed self correcting control model are shown in Fig 6.

From Fig 6, it can be seen that under the control of the proposed self correcting model, the table tennis wheelchair smoothly cuts into the circular trajectory from the initial position, and then smoothly moves along the circular trajectory. Within the time range of t = 0.5 ~ 1.0s, there is a slipping effect between the ground and the wheelchair, but under the control of the self correcting model, the movement trajectory of the wheelchair remains stable. In Fig 6, the table tennis wheelchair travels through a certain distance from the origin position before entering the expected circular trajectory, which is also a consideration of the initial disturbance that may occur in the wheelchair after adding electrical control in the future. After such processing, the integrity of its circular trajectory can be ensured.

**Fig 7:**
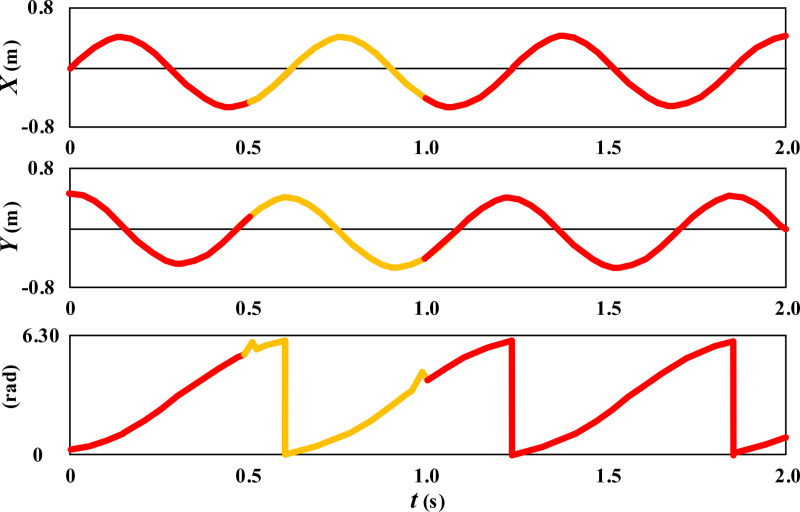
Dimensionality expansion results of circular movement posture of wheelchair.

Expand the circular movement posture of the wheelchair in Fig 5 along the time axis, as shown in Fig 7.

From the results in Fig 7, it can be seen that during circular movement, the wheelchair maintains sine and cosine variations in both the X and Y directions. Although the slip of 0.5-1s has a slight impact, the self correcting control model can provide the fastest correction, ensuring the stability of the wheelchair movement trajectory. During circular movement, the angle  φ of the wheelchair varies periodically between 0–2 π. The 0.5-1s slip caused a trend of angle adjustment for the wheelchair, but the self correcting model was able to provide timely correction, ensuring the stability of the wheelchair angle.

**Fig 8:**
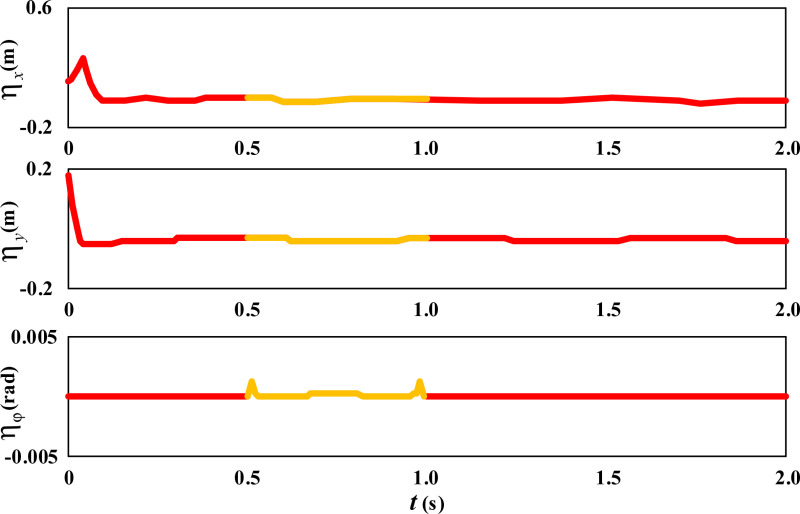
Dimensionality expansion results of circular movement error of wheelchair.

Further observe the error changes in the movement trajectory of the wheelchair during the movement process, as shown in Fig 8.

From Fig 8, it can be seen that there are slight fluctuations in the trajectory error curves of the wheelchair in both the X and Y directions during circular movement. The largest fluctuation occurs during the period when the wheelchair cuts into the circular trajectory from the initial point. The angle error $\eta_{\varphi }$ of the wheelchair fluctuates slightly between 0.5 and 1 second, which is the result of the wheelchair continuously correcting the angle to cope with slipping. Under the proposed control algorithm, the wheelchair smoothly travels on the expected trajectory by adjusting and continuously reducing angle and trajectory errors, achieving the desired control effect.

## 5 Discussion

Other methods can also be used to control the movement trajectory of the table tennis wheelchair. Among these methods, PID method and Particle Swarm Optimization (PSO) method were chosen. Apply these two methods to control the linear and circular movement of table tennis wheelchairs, as well as to handle slip interference, in order to form and compare the proposed self correction methods. The reason for choosing PID method and PSO method as reference methods is that PID method is a classic method in the field of control and has the widest range of applications. The PSO method has good specificity in trajectory control optimization, and there are also significant similarities between table tennis wheelchair slip control and trajectory optimization.

**Fig 9:**
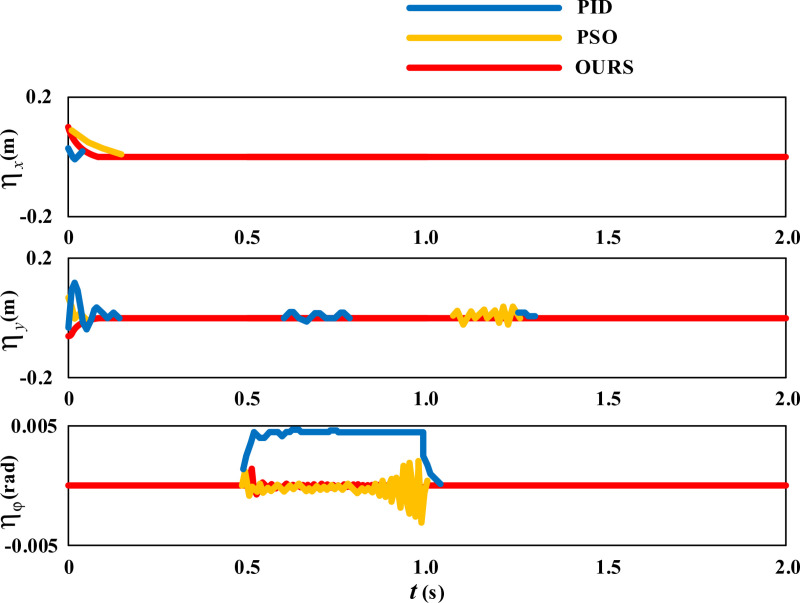
Error Comparison of Wheelchair Linear Movement Trajectory.

Firstly, let’s observe the control effects of the three methods on the straight-line movement of wheelchairs. The error comparison results of the linear movement trajectory are shown in Fig 9.

In Fig 9, the blue line represents the control effect of the PID method, the orange line represents the control effect of the PSO method, and the red line represents the control effect of the proposed self correction method. From the variation of the error curve of the movement trajectory in the X direction, it can be seen that the PID method has some fluctuations, while the PSO method converges slightly slower. From the variation of the error curve of the Y-direction movement trajectory, it can be seen that the PID method has a longer fluctuation period and larger amplitude. From the variation curve of angle error $\eta_{\varphi }$, it can be seen that when slippage occurs within the range of t = 0.5 ~ 1.0s, the fluctuation amplitude of the PID method is large, and the vibration of the PSO method is relatively severe. In contrast, under the control of the proposed self correction method, the movement trajectory error of the wheelchair is minimized.

The statistical results of the changes in the three sets of variables in Fig 9 are shown in Table 1

**Table 1:** Statistical results of changes in three sets of variables in Fig 9.

Method	Parameter	Maximum	Minimum	Average
**PID**	$\eta_{x}$	0.034	0	0.0009
**PSO**		0.112	-0.002	0.0034
**OURS**		0.113	0	0.0028
**PID**	$\eta_{y}$	0.135	-0.039	0.0017
**PSO**		0.089	-0.005	0.0024
**OURS**		0	-0.083	0.0015
**PID**	$\eta_{\varphi }$	0.00499	0	0.00133
**PSO**		0.00248	-0.00312	-0.00011
**OURS**		0.00165	-0.00078	0.00031

**Fig 10:**
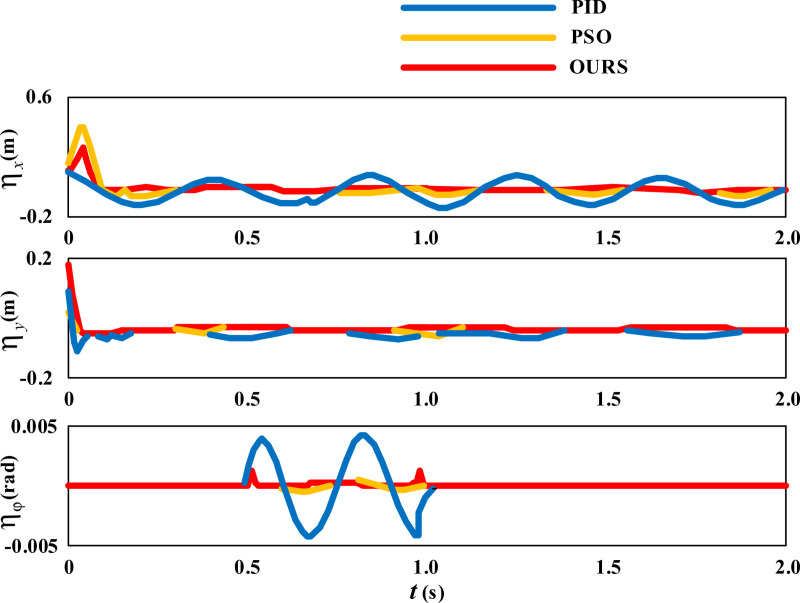
Error Comparison of Wheelchair Circular Movement Trajectory.

Next, let’s observe the control effects of the three methods on the circular movement of the wheelchair. The error comparison results of the circular movement trajectory are shown in Fig 10.

In Fig 10, the blue line represents the control effect of the PID method, the orange line represents the control effect of the PSO method, and the red line represents the control effect of the proposed self correction method. From the variation of the error curve of the movement trajectory in the X direction, it can be seen that the overall stationarity of PSO method and self correction method is close, while the fluctuation amplitude of PID method is large and continues throughout the entire movement process. From the variation of the error curve of the Y-direction movement trajectory, it can be seen that the overall stationarity of PSO method and self correction method is close. The fluctuation of the PID method persists throughout the entire movement process, but the amplitude of the fluctuation is smaller than in the X direction. From the variation curve of angle error $\eta_{\varphi }$, it can be seen that when slippage occurs within the range of t = 0.5 ~ 1.0s, the fluctuation amplitude of the PID method is large. In contrast, under the control of the proposed self correction method, the movement trajectory error of the wheelchair is minimized.

The statistical results of the changes in the three sets of variables in Fig 10 are shown in Table 2.

**Table 2:** Statistical results of changes in three sets of variables in Fig 10.

Method	Parameter	Maximum	Minimum	Average
**PID**	$\eta_{x}$	0.108	-0.169	-0.0255
**PSO**		0.341	-0.087	-0.0067
**OURS**		0.235	0	0.0019
**PID**	$\eta_{y}$	0.093	-0.121	-0.0035
**PSO**		0.045	-0.041	-0.0016
**OURS**		0.191	-0.022	-0.0013
**PID**	$\eta_{\varphi }$	0.00462	-0.00471	0.00128
**PSO**		0.00017	-0.00025	-0.00012
**OURS**		0.00151	-0.00013	0.00019

## 6 Conclusion

Wheelchairs are important equipment in table tennis for people with disabilities, and their effective control of the movement process directly affects the athlete’s positioning and strength, ultimately affecting their competition results. The wheelchair will move according to the athlete’s posture and movement trend, but the movement process may be affected by interference such as slipping, resulting in significant deviations from the expected trajectory. Therefore, systematic research has been carried out on the control problem of table tennis wheelchairs. Starting from the body structure of the table tennis wheelchair, a kinematic model of the wheelchair was constructed, and the relationship between the body motion and the rotation of each wheel was established. Considering the impact of slipping on each wheel, a trajectory error model for a table tennis wheelchair was derived. A self correcting control model based on compensation mechanism was designed to address the impact of slip and other disturbances, and the control rate was derived. Through proof, it has been theoretically confirmed that the self correcting model is Lyapunov stable. During the simulation experiment, the rationality of the wheelchair kinematic model and the effectiveness of the self correcting control model were verified from two angles: linear movement and circular movement. In the presence of slip interference, the self correcting model can still control the wheelchair to move on a predetermined trajectory and maintain stability. Further comparative experiments have verified the control effectiveness of the proposed self correction model, which is superior to PID and PSO methods.
